# Prevalence of dyslipidemia among persons with type 2 diabetes in Africa: a systematic review and meta-analysis

**DOI:** 10.1097/MS9.0000000000002122

**Published:** 2024-05-06

**Authors:** Emmanuel Ekpor, Dorothy Addo-Mensah, Samuel Akyirem

**Affiliations:** a School of Nursing and Midwifery, University of Ghana, Legon; b Christian Health Association of Ghana, Accra, Ghana; c School of Nursing, The University of North Carolina at Chapel Hill, Chapel Hill, NC; d Yale School of Nursing, Yale University, New Haven, CT

**Keywords:** abnormal lipids, Africa, dyslipidemia, systematic review, type 2 diabetes

## Abstract

**Background:**

Dyslipidemia is an established cardiovascular risk factor in persons with type 2 diabetes (T2D), yet the extent to which these conditions co-occur in Africa is unknown. This systematic review and meta-analysis aimed to determine the prevalence of dyslipidemia among persons with T2D in Africa.

**Methods:**

Medline, Embase, Global Health, Scopus, and Web of Science were searched on 5 December 2023. This review adhered to the PRISMA guidelines and was prospectively registered on PROSPERO. The prevalence data for dyslipidemia was pooled through a random-effects meta-analysis. The authors assessed heterogeneity and publication bias using *I*^*2*^ statistics and Egger’s test, respectively.

**Results:**

Our search identified 8035 records, of which 60 articles involving 20 034 individuals with T2D were included in this review. The pooled prevalence of dyslipidemia recorded 38.6% (95% CI: 34.1–43.4) for high TC (≥ 5.2 mmol/l), 52.7% (95% CI: 44.2–61.1) for high low-density lipoprotein cholesterol (LDL-C) (≥ 2.6 mmol/l), 43.5% (95% CI: 37.1–50.0) for low high-density lipoprotein cholesterol (HDL-C) (< 1.0 mmol/l in men and less than 1.3 mmol/l in women), and 37.4% (95% CI: 32.2–42.9) for high triglycerides (TG) (≥ 1.7 mmol/l). Subgroup analysis based on gender indicated a notably higher prevalence of dyslipidemia among females compared to males.

**Conclusion:**

Dyslipidemia is prevalent among persons with T2D in Africa. This highlights the need for early screening, diagnosis, and management of dyslipidemia to mitigate the risk of cardiovascular complications in this population.

## Introduction

Highlights

- There is a high prevalence of dyslipidemia among persons with type 2 diabetes (T2D) in Africa, with rates ranging from 37.4% for high triglycerides (TG) to 52.7% for high low-density lipoprotein cholesterol (LDL-C).
- Females exhibited a high prevalence of dyslipidemia compared to males.
- Persons with T2D in Africa may be at high risk of cardiovascular complications.

The global prevalence of diabetes is increasing, with Africa projected to bear a disproportionately high burden of this metabolic disorder in the forthcoming years. With 24 million people living with diabetes in Africa as of 2021, the continent is estimated to have a 134% increase in diabetes cases by the year 2045^1^. Type 2 diabetes (T2D) accounts for 90% of all diabetes cases and ranks among the leading causes of death in the African continent^2^. T2D is characterized by chronic hyperglycaemia, which gives rise to a range of microvascular and macrovascular complications, significantly impacting both quality of life and life expectancy^3^.

Dyslipidemia has emerged as an important clinical abnormality in persons with T2D^4^. In fact, research has established a strong link between dyslipidemia and T2D, with the term “diabetic dyslipidemia” used as a descriptor for the lipid abnormalities in persons with T2D. This condition is characterized by disturbances in serum lipid profiles such as high total cholesterol (TC), low-density lipoprotein cholesterol (LDL-C), triglycerides (TG), or low concentrations of high-density lipoprotein cholesterol (HDL-C)^4^. Across different regions worldwide, a high prevalence of dyslipidemia among persons with T2D has been recorded, with studies reporting rates exceeding 65%^5–7^. Both T2D and dyslipidemia are independent risk factors for cardiovascular diseases (CVD), and their coexistence further increases the risk of composite CVD outcomes and coronary artery disease events in persons with T2D^8^. Additionally, dyslipidemia has been implicated in compromising health-related quality of life among persons with T2D^9^.

Indeed, the lipid profile of persons with T2D is an important indicator of their health. Hence, clinical practice guidelines recommend regular monitoring and management of dyslipidemia in individuals with T2D. The America Diabetes Association (ADA) recommends initiating statin (a cholesterol-lowering drug) for T2D patients with additional cardiovascular risk factors^10^. However, despite the increasing prevalence of CVD and its associated risk factors in Africa^11,12^, studies have revealed that T2D patients in the region often do not receive the recommended statin therapy for primary prevention of CVD^13,14^.

In Africa, the extent of dyslipidemia among persons with T2D remains inadequately understood. This knowledge gap may undermine efforts to enact preventive strategies against CVD within this vulnerable patient population. Therefore, this review aimed to determine the prevalence of dyslipidemia among persons with T2D in Africa by synthesizing the results of relevant studies across the continent. The findings of this review hold significant implications for developing evidence-based interventions and policies aimed at improving the management of dyslipidemia and reducing the risk of CVD in persons with T2D.

## Methods

### Search strategy

The Preferred Reporting Items for Systematic Reviews and Meta-Analyses (PRISMA, Supplemental Digital Content 3, http://links.lww.com/MS9/A462) guidelines was followed in reporting this review^15^. Also, the AMSTAR 2 checklist, Supplemental Digital Content 4, http://links.lww.com/MS9/A463 was completed to evaluate the study’s quality (Supplementary Digital Content 1, http://links.lww.com/MS9/A460). The protocol for this review was developed and registered in the PROSPERO International Prospective Register of Systematic Reviews.

We searched Medline, Embase, Global Health, Scopus, and Web of Science to identify all relevant articles for this review. This was supplemented with additional searches on African Journals Online (AJOL) and the reference list of all relevant articles. The search was executed on 5 December 2023, focusing on studies published from 2001 to 2023. Our search strategy encompassed terms in relation to “dyslipidemia”, “type 2 diabetes” and a list of all 54 countries in Africa. Controlled vocabulary and keywords to the search terms were incorporated, with the Boolean operators ‘OR’ and ‘AND’ applied appropriately. The full search strategy is available in Supplementary Digital Content 2, Table S1, http://links.lww.com/MS9/A461.

### Inclusion and exclusion criteria

We included cross-sectional studies reporting the prevalence of serum lipid abnormalities (i.e. high TC, high LDL-C, low HDL-C, and high TG) among persons with T2D in Africa, or studies providing adequate data for computation. To ensure consistency and comparability across studies, we exclusively considered studies utilizing the National Cholesterol Education Program (NCEP) guidelines as the benchmark for defining abnormal lipid levels^16^. Specifically, this entailed levels of TC greater than or equal to 5.2 mmol/l, HDL-C less than 1 mmol/l for men and less than <1.3 mmol/l for women, TG greater than or equal to 1.7 mmol/l, and LDL-C greater than or equal to 2.6 mmol/l (or their equivalent values in mg/dl).

The exclusion criteria for this review encompassed commentaries, editorials, abstracts, reviews, and non-English articles. Additionally, we excluded studies that involved participants younger than 18 years, along with studies with a sample size of less than 100 individuals with T2D. In cases where studies were published in multiple reports, we selected only one with the largest sample size.

### Study selection and data extraction

The articles retrieved from our search were imported into Endnote 20 to remove duplicate records. Subsequently, the remaining articles were uploaded onto Rayyan for a two-stage screening process by two reviewers. Initial screening was conducted based on the title and abstract, followed by a more comprehensive evaluation of the full text for eligible articles. Any disagreements arising during the screening process were resolved through discussion and consensus.

We extracted relevant information from the studies using a standardized data extraction matrix in Microsoft excel. The information gathered included the first author’s name, publication year, country, Africa region, sample size, gender distribution, participant’s average age, years lived with T2D, and forms of abnormal lipid profiles investigated, along with their respective prevalence rates.

### Quality assessment

The quality assessment of the included studies was conducted using the Joanna Briggs Institute (JBI) 9-item checklist tailored for prevalence studies^17^. This checklist comprises nine critical questions, each with response options including “Yes,” “No,” “Unclear,” and “Not applicable.” Two authors performed the quality assessment, rating studies with 7–9, 5–6, and less than 5 “yes” as having low, moderate, and high risk of bias, respectively.

### Data analysis

The meta package in R statistical software was used for the meta-analysis. A logit‐transformation was performed as suggested by Warton and Hui^18^, while the 95% CI of the prevalence estimates were calculated using the Clopper–Pearson interval. A DerSimonian-Laird’s random-effects model was used to estimate the pooled prevalence of the abnormal lipids. Heterogeneity was assessed using the I² statistic, where values of 25%, 50%, and 75% represented low, moderate, and high heterogeneity, respectively^19^. Subgroup analysis was performed to identify variation in prevalence estimates based on geographical regions in Africa and gender. Additionally, we performed sensitivity analysis by excluding studies of low quality from the meta-analysis. The presence of publication bias was evaluated using the Egger’s test, with *P* less than 0·05 indicating significant publication bias^20^.

## Results

### Search results

**Figure 1:**
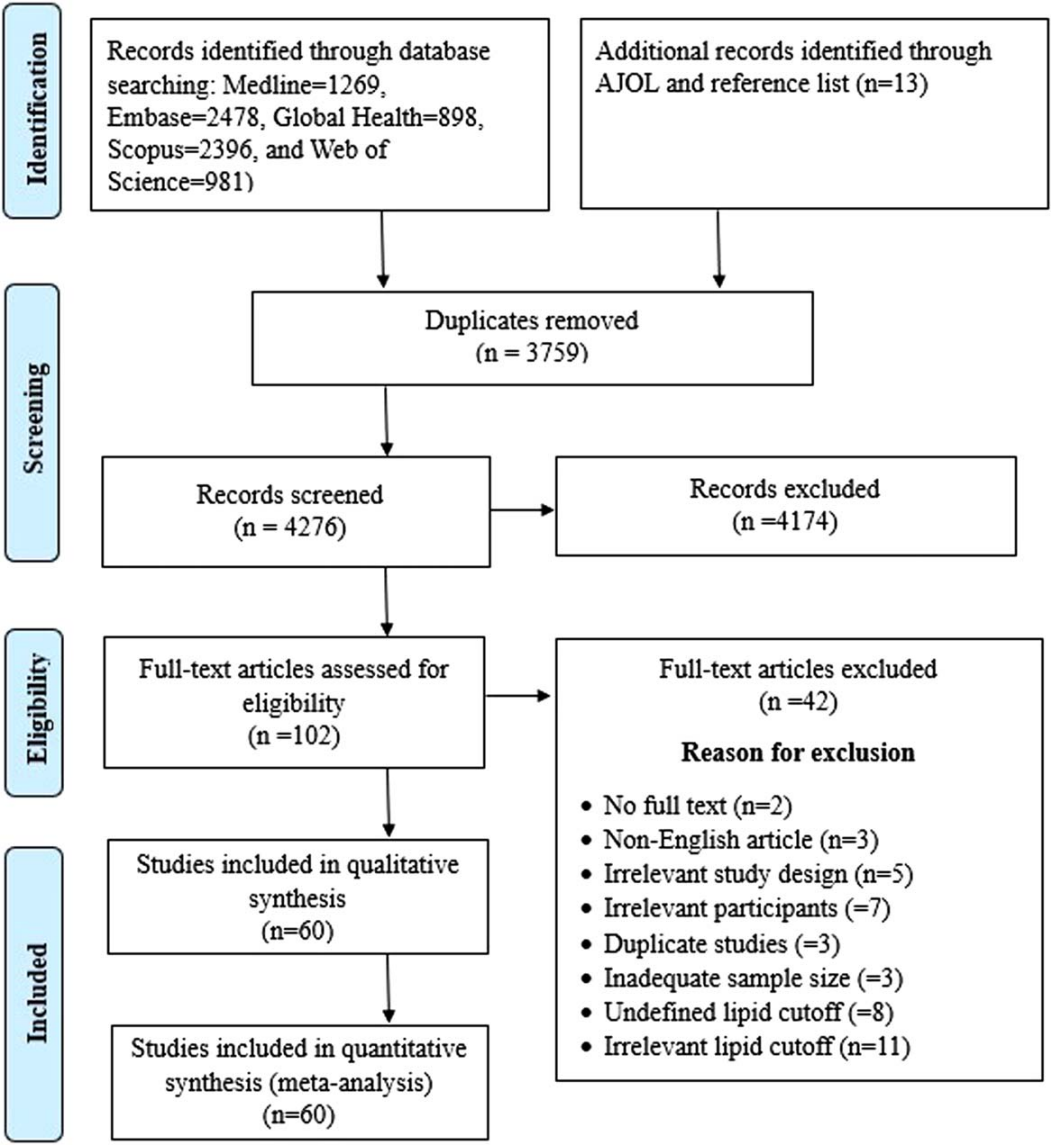
PRISMA flow chart summarizing the article selection process. AJOL, African Journals Online; PRISMA, preferred reporting items for systematic reviews and meta-analyses.

Our search yielded a total of 8035 references, comprising 8022 records sourced from major databases (Medline, Embase, Global Health, Scopus, and Web of Science) and thirteen from other supplemental searches on AJOL and reference lists of relevant articles. After removing duplicate records, we screened 4276 articles based on their titles and abstracts. Following this, the full texts of 102 articles underwent thorough assessment, ultimately resulting in the inclusion of 60 articles in this review (Fig. 1).

### Characteristics of included studies

The articles included in this review were published between 2004 and 2023, with over 70% published after 2014. Recruitment of the T2D participants occurred across various healthcare facilities. With the exception of Sobngwi *et al*.^21^‘s study, which involved participants from multiple countries in sub-Saharan Africa, all other studies focused on a single country. Nigeria was the most prominently represented, with 14 articles^22–35^. This was followed by Ghana (*n*=13)^36–48^, Ethiopia (*n*=12)^49–60^, South Africa (*n*=5)^61–65^, Kenya (*n*=3)^66–68^, Sudan (*n*=3)^69–71^, Eritrea (*n*=2)^72,73^, and one article each from Algeria^74^, Egypt^75^, Morocco^76^, Somalia^77^, Tanzania^78^, Uganda^79^, and Zambia^80^. In total, 20 034 participants were studied, with sample sizes ranging from 100 to 2352. Gender distribution was reported in 57 articles, indicating a female representation of 57.8%. The mean age of participants ranged from 49.6 to 63.3 years, and the mean diabetes duration ranged from 4 to 12.1 years. Full details of the characteristics of the included studies are presented in Supplementary Digital Content 2, Table S2, http://links.lww.com/MS9/A461.

### Prevalence of dyslipidemia

**Figure 2:**
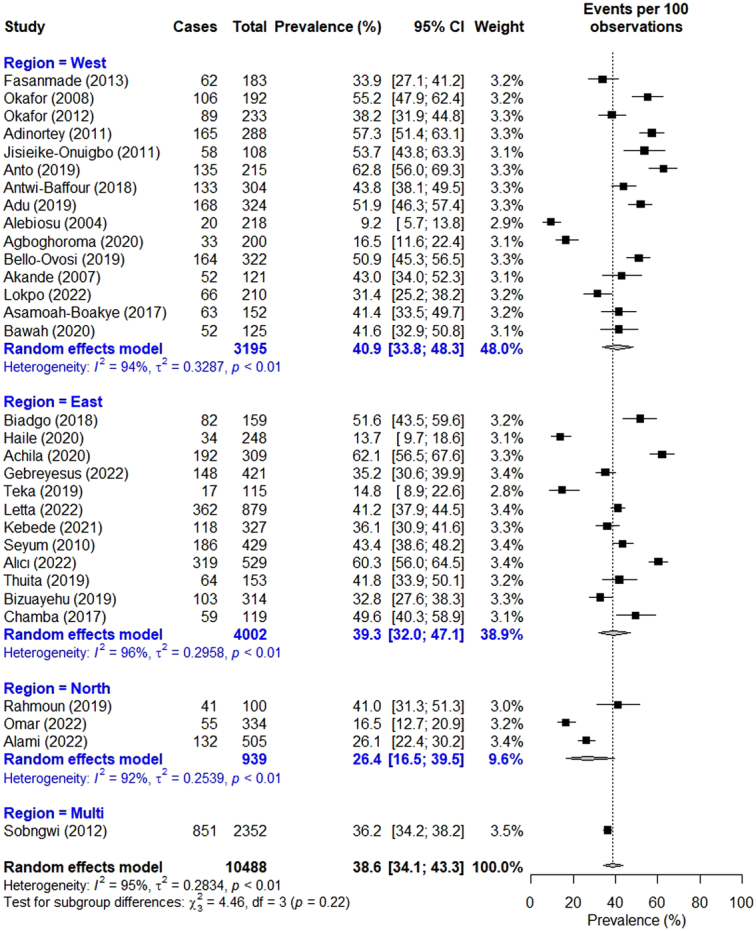
Forest plot for pooled prevalence of high total cholesterol in persons with type 2 diabetes.

Thirty-one studies provided data on the prevalence of high TC, with the rate ranging from 9.2% in Nigeria^34^ to 62.8% in Ghana^38^. The pooled prevalence of high TC recorded 38.6 (95% CI: 34.1–43.4) (Fig. 2). The prevalence varied across regions, with the rate recording 26.4% (95% CI: 16.5–39.5) in the North, 39.3% (95% CI: 32.0–47.1) in the East, and 40.9% (95% CI: 33.8–48.3) in the West. Based on gender, the prevalence rate recorded 37.2% (95% CI: 28.8–46.4) in males and 51.0% (95% CI: 42.8–59.2) in females.

**Figure 3:**
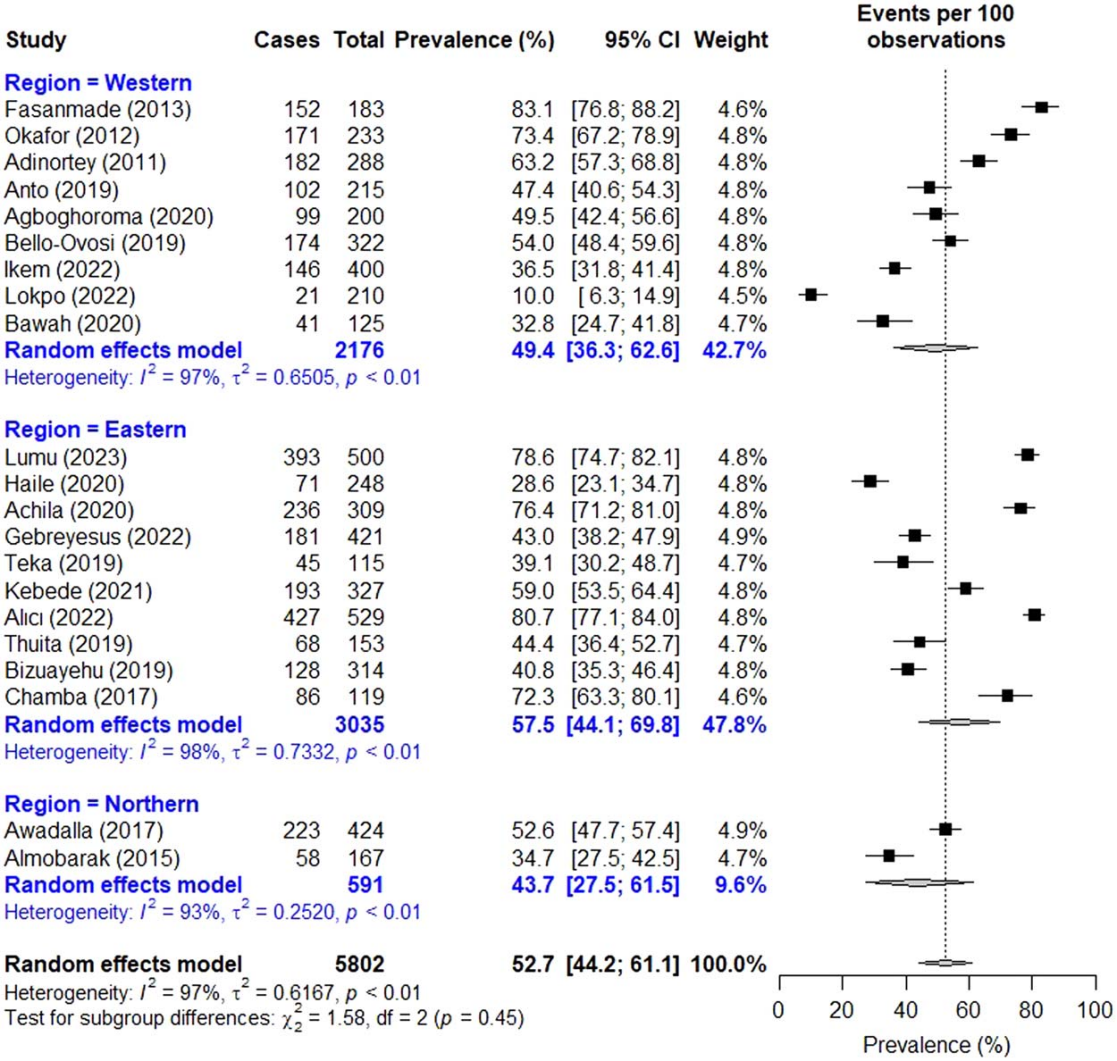
Forest plot for pooled prevalence of high low-density lipoprotein cholesterol in persons with type 2 diabetes.

Across 21 studies, the prevalence of high LDL-C ranged from 10.0% in Ghana^42^ to 83.1% in Nigeria^22^. The overall prevalence of high LDL-C recorded 52.7% (95% CI: 44.2–61.1) (Fig. 3). By region, the prevalence rate was 43.7% (95% CI: 27.5–61.5) in the North, 49.4% (95% CI: 36.3–62.6) in the West, and 57.5% (95% CI: 44.1–69.8) in the East. In terms of gender, the prevalence rate was 48.9% (95% CI: 30.6–67.5) in males and 63.0% (95% CI: 40.3–81.1) in females.

**Figure 4:**
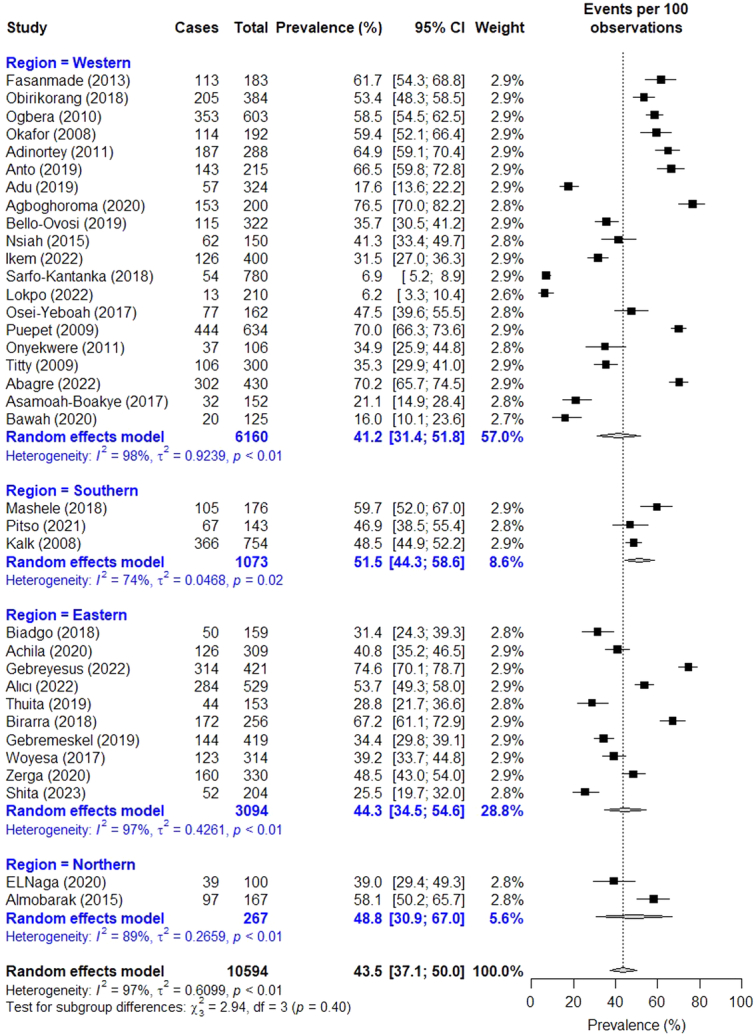
Forest plot for pooled prevalence of low high-density lipoprotein cholesterol in persons with type 2 diabetes.

The prevalence rate of low HDL-C (*n*=35) ranged from 6.2% in Ghana^42^ to 76.5% in Nigeria^26^. The pooled prevalence across studies recorded 43.5% (95% CI: 37.1–50.0) (Fig. 4). There was a regional variation in low HDL-C prevalence, recording 41.2% (95% CI: 31.4–51.8) in the West, followed by 44.3% (95% CI: 34.5–54.6) in the East, 48.8% (95% CI: 30.9–67.0) in the North, and 51.5% (95% CI: 44.3–58.6) in the South. The prevalence estimate varied according to gender, with males recording a rate of 37.0% (95% CI: 29.8–44.8) while females recorded 45.4% (95% CI: 36.3–54.9).

**Figure 5:**
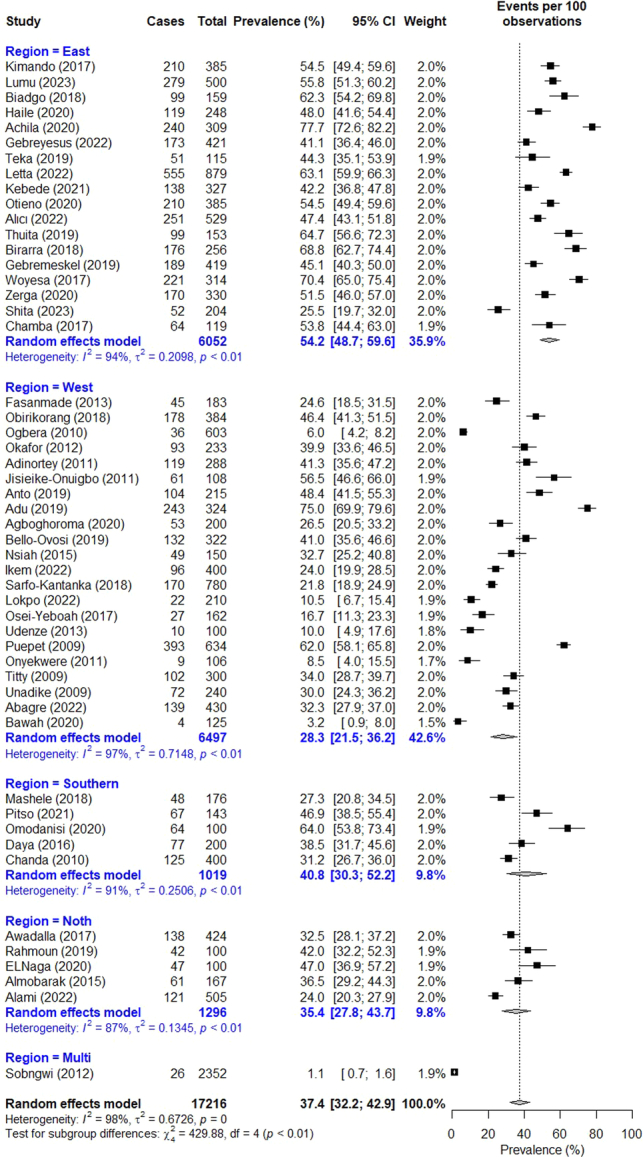
Forest plot for pooled prevalence of high triglycerides in persons with type 2 diabetes.

The prevalence of high TG as reported by 51 studies ranged from 1.1% in Sobngwi *et al*.^21^’s multi country study to 77.7% in a study conducted in Eritrea^72^. The pooled prevalence of high TG was recorded 37.4% (95% CI: 32.2–42.9). (Fig. 5) By region, West Africa recorded the lowest prevalence of 28.3% (95% CI: 21.5–36.2). This was followed by 35.4% (95% CI: 27.8% 43.7%) in the North, 40.8% (95% CI: 30.3–52.2) in the South, and 54.2% (95% CI: 48.7–59.6) in the East. Based on gender, the prevalence rate recorded 39.3% (95% CI: 32.1–46.9) in males and 44.2% (95% CI: 37.2–51.4) in females.

### Quality assessment and of risk of bias

Full detail of the quality assessment is provided in the Supplementary Digital Content 2, Table S3, http://links.lww.com/MS9/A461. The quality score of the included studies ranged from 2 to 9, with a mean score of 6.5. Fifty-two studies had a quality score of 5 and above, indicating low-medium risk of bias. Sensitivity analysis on the basis of excluding articles identified with a high risk of bias (n=8)^24,32–34,63,69,70,74^, revealed a marginal difference in the overall prevalence of abnormal lipid profiles (Supplementary Digital Content 2, Figure S1–S4, http://links.lww.com/MS9/A461).

Across the various lipid parameters assessed, only the funnel plot for studies on high level of TG showed an asymmetrical distribution, indicating the presence of publication bias (Supplementary Digital Content 2, Figure S5–S8, http://links.lww.com/MS9/A461). This was statistically confirmed with Egger’s test (*P* = 0.009). The Egger’s test for high TC (*P* = 0.621), high LDL-C (*P* = 0.473), and low HDL-C (*P* = 0.024) showed no evidence of publication bias.

## Discussion

To the best of our knowledge, this study marks a pioneering effort in systematically reviewing and synthesizing the prevalence of dyslipidemia among individuals with T2D in Africa. Our findings revealed a high prevalence of dyslipidemia, with rates ranging from 37.4% for high TG to 52.7% for high LDL-C. While the prevalence of these lipid abnormalities varied across different regions of Africa, substantial rates were consistently observed. This underscores the profound burden of dyslipidemia, which transcends geographical boundaries throughout the African continent. Moreover, we identified that females consistently had a high prevalence of all lipid abnormalities compared to males, highlighting the need for gender-specific approaches in managing dyslipidemia among individuals with T2D in Africa.

In the realm of patient care, dyslipidemia is commonly defined by the interplay of multiple lipid variables. However, as highlighted in a previous meta-analysis^81,82^, we reported the prevalence of single components of dyslipidemia because most of the studies included in this review did not provide comprehensive data on all lipid parameters. Regardless, our results dispel any doubts regarding the substantial burden of dyslipidemia in Africa. Notably, previous meta-analysis among the general population in Africa have also reported a high prevalence of dyslipidemia^81^. However, it is worth noting that the rates across the various lipid profiles were lower compared to our results. This emphasizes the importance of addressing dyslipidemia as a significant comorbidity of T2D in African populations.

The high prevalence of dyslipidemia among persons with T2D may be due to several factors. Firstly, T2D often coexists with other health conditions, such as hypertension and obesity, which are also risk factors for dyslipidemia. Considering the high prevalence of hypertension (60.8%)^83^ and both overweight and obesity (61.4%)^12^ in persons with T2D in Africa, it is plausible that the clustering of these metabolic abnormalities may further exacerbate the risk of dyslipidemia in this population. Moreover, studies in Africa have reported a high consumption of processed foods rich in saturated fats as well as the adoption of a sedentary lifestyle^84^, all of which has been established as a significant contributor of dyslipidemia. Additionally, Reiger *et al.*^85^ has revealed a concerning lack of awareness regarding dyslipidemia among the African population. This lack of awareness could lead to underdiagnosis and inadequate management of dyslipidemia among individuals with T2D in Africa.

With the high prevalence of dyslipidemia among persons with T2D in Africa, there is a heightened risk of cardiovascular complications within this population. This underscores the importance of routine screening and monitoring of lipid profiles among persons with T2D in Africa to enable early detection and timely intervention for cardiovascular complications. Healthcare providers should adopt a comprehensive approach to diabetes care that includes appropriate interventions for addressing dyslipidemia. However, while clinical guidelines recommend the prescription of lipid-lowering medication as a pharmacological therapy of dyslipidemia when warranted^10^, these drugs are often inaccessible or unaffordable across the African continent^86^. Addressing this challenge requires concerted efforts from policymakers to overcome barriers to accessing essential medications for dyslipidemia management in Africa. Furthermore, there is a critical need for health education and awareness campaigns aimed at empowering individuals with knowledge about dyslipidemia, its associated risks, and the importance of proactive management strategies.

### Strength and limitations

The major strength of this review lies in its novelty, offering a valuable benchmark for future research. Also, the inclusion of only cross-sectional studies in the meta-analysis ensured the robustness of our findings, given their appropriateness for prevalence studies. However, this study has some limitations which are worth acknowledging. Firstly, the studies included in the meta-analysis exhibited a significant level of heterogeneity which may influence our prevalence estimate. Additionally, the lack of comprehensive data on all lipid parameters in the included studies limited the depth of our analysis. Future studies should aim to collect data on all lipid profiles to better characterize dyslipidemia among individuals with T2D in Africa. Furthermore, the level of lipid abnormalities in this study was based exclusively on the NCEP’s cutoff. Hence, our prevalence estimates may not entirely reflect the burden of dyslipidemia among persons with T2D in Africa.

## Conclusions

This review identified a high prevalence of dyslipidemia among persons with T2D in Africa, highlighting the need for early screening, diagnosis, and management of dyslipidemia to mitigate the risk of cardiovascular complications in this population. Further research focusing on region-specific risk factors and interventions is needed to inform targeted preventive measures and improve clinical outcomes among persons with T2D in Africa.

## Ethical approval

Not applicable

## Consent

Informed consent was not required for this review.

## Sources of funding

No funding was received to undertake this project

## Author contribution

E.E.: conceptualization, data curation, methodology, investigation, formal analysis, validation, software, visualization, resources, project administration, writing original draft, and writing review and editing. D.A.-M.: data curation, methodology, investigation, formal analysis, validation, writing review and editing. S.A.: methodology, investigation, formal analysis, validation, software, visualization, writing review and editing, and supervision. All authors revised and approved the final manuscript.

## Conflict of interest disclosure

The authors have declared that no competing interests exist.

## Research registration unique identifying number (UIN)

PROSPERO, https://www.crd.york.ac.uk/prospero/display_record.php?RecordID=491237 Registration ID: CRD42023491237.

## Guarantor

Emmanuel Ekpor.

## Data availability statement

All data for this review can be accessed in this manuscript and its supporting information files.

## Provenance and peer review

No invitation was received for this manuscript.

## Supplementary Material

SUPPLEMENTARY MATERIAL
