# Supplementary material for: Prevalence of dyslipidemia among persons with type 2 diabetes in Africa: a systematic review and meta-analysis
Source: Ann Med Surg (Lond). 2024 May 6;86(6):3468–77. doi: 10.1097/MS9.0000000000002122 (PMC11152840; doi:10.1097/MS9.0000000000002122)
Supplement: SUPPLEMENTARY MATERIAL [file ms9-86-3468-s002.pdf]

Prevalence of dyslipidemia among persons with type 2 diabetes in Africa: a systematic review and meta-analysis

Table S1. Database search strategies

Concept	Search strings
**Medline**	
#1 Dyslipidemia	exp Dyslipidemias/ or exp Hyperlipidemias/ or exp Hypercholesterolemia/ or exp Hypertriglyceridemia/ or (Dyslipidemia* or dyslipid* or hyperlipidemia* or hyperlipid* or "lipid disorder*" or lipid* OR "lipid profile" or hypercholesterolemia or hypercholesterol* or hypertriglyceridemia or hypertriglycerid*).ti,ab.
#2 Type 2 diabetes	exp Diabetes Mellitus/ or exp Diabetes Mellitus, Type 2/ or (diabetes or "type 2 diabetes" or "non-insulin-dependent diabetes mellitus" or NIDD or T2D or T2DM).ti,ab.
#3 Africa	exp Africa/ or (Africa or Algeria or Angola or Benin or Botswana or "Burkina Faso" or Burundi or "Cabo Verde" or Cameroon or "Central African Republic" or Chad or Comoros or "Democratic Republic of the Congo" or "Republic of the Congo" or "Cote d'Ivoire" or Djibouti or Egypt or "Equatorial Guinea" or Eritrea or Eswatini or Ethiopia or Gabon or Gambia or Ghana or Guinea or Guinea-Bissau or Kenya or Lesotho or Liberia or Libya or Madagascar or Malawi or Mali or Mauritania or Mauritius or Morocco or Mozambique or Namibia or Niger or Nigeria or Rwanda or "Sao Tome and Principe" or Senegal or Seychelles or "Sierra Leone" or Somalia or "South Africa" or "South Sudan" or Sudan or Tanzania or Togo or Tunisia or Uganda or Zambia or Zimbabwe).ti,ab.
#4	#1 and #2 and #3
#5	#4 limit to yr="2001 - 2023"
**Embase**	
#1 Dyslipidemia	exp dyslipidemia/ or exp hyperlipidemia/ or exp hypercholesterolemia/ or exp hypertriglyceridemia/ or (Dyslipidemia* or dyslipid* or hyperlipidemia* or hyperlipid* or "lipid disorder*" or lipid* OR "lipid profile" or hypercholesterolemia or hypercholesterol* or hypertriglyceridemia or hypertriglycerid*).ti,ab.
#2 Type 2 diabetes	exp diabetes mellitus/ or exp non insulin dependent diabetes mellitus/ or (diabetes or "type 2 diabetes" or "non-insulin-dependent diabetes mellitus" or NIDD or T2D or T2DM).ti,ab.
#3 Africa	exp Africa/ or (Africa or Algeria or Angola or Benin or Botswana or "Burkina Faso" or Burundi or "Cabo Verde" or Cameroon or "Central African Republic" or Chad or Comoros or "Democratic Republic of the Congo" or "Republic of the Congo" or "Cote d'Ivoire" or Djibouti or Egypt or "Equatorial Guinea" or Eritrea or Eswatini or Ethiopia or Gabon or Gambia or Ghana or Guinea or Guinea-Bissau or Kenya or Lesotho or Liberia or Libya or Madagascar or Malawi or Mali or Mauritania or Mauritius or Morocco or Mozambique or Namibia or Niger or Nigeria or Rwanda or "Sao Tome and Principe" or Senegal or Seychelles or "Sierra Leone" or Somalia or "South Africa" or "South Sudan" or Sudan or Tanzania or Togo or Tunisia or Uganda or Zambia or Zimbabwe).ti,ab.
#4	#1 and #2 and #3
#5	#4 limit to yr="2001 - 2023"

Global health

#1 Dyslipidemia	exp lipid metabolism disorders/ or exp hyperlipaemia/ or exp hypercholesterolaemia/ or exp hypertriglyceridaemia/ or (Dyslipidemia* or dyslipid* or hyperlipidemia* or hyperlipid* or "lipid disorder*" or "lipid metabolism disorders" or lipid* or "lipid profile" or hypercholesterolemia or hypercholesterol* or hypertriglyceridemia or hypertriglycerid*).ti,ab.
#2 Type 2 diabetes	exp diabetes mellitus/ or exp type 2 diabetes/ or (diabetes or "type 2 diabetes" or "non-insulin-dependent diabetes mellitus" or NIDD or T2D or T2DM).ti,ab.
#3 Africa	exp Africa/ or (Africa or Algeria or Angola or Benin or Botswana or "Burkina Faso" or Burundi or "Cabo Verde" or Cameroon or "Central African Republic" or Chad or Comoros or "Democratic Republic of the Congo" or "Republic of the Congo" or "Cote d'Ivoire" or Djibouti or Egypt or "Equatorial Guinea" or Eritrea or Eswatini or Ethiopia or Gabon or Gambia or Ghana or Guinea or Guinea-Bissau or Kenya or Lesotho or Liberia or Libya or Madagascar or Malawi or Mali or Mauritania or Mauritius or Morocco or Mozambique or Namibia or Niger or Nigeria or Rwanda or "Sao Tome and Principe" or Senegal or Seychelles or "Sierra Leone" or Somalia or "South Africa" or "South Sudan" or Sudan or Tanzania or Togo or Tunisia or Uganda or Zambia or Zimbabwe).ti,ab.
#4	#1 and #2 and #3
#5	Limit #4 to yr="2001 - 2023"

Scopus

#1 Dyslipidemia	TITLE-ABS-KEY (Dyslipidemia* OR dyslipid* OR hyperlipidemia* OR hyperlipid* OR "lipid disorder*" OR "lipid metabolism disorders" OR lipid* OR "lipid profile" OR hypercholesterolemia OR hypercholesterol* OR hypertriglyceridemia OR hypertriglycerid*)
#2 Type 2 diabetes	TITLE-ABS-KEY (Diabetes OR "type 2 diabetes" OR "non-insulin-dependent diabetes mellitus" OR NIDD OR T2D OR T2DM)
#3 Africa	TITLE-ABS-KEY (Africa OR Algeria OR Angola OR Benin OR Botswana OR "Burkina Faso" OR Burundi OR "Cabo Verde" OR Cameroon OR "Central African Republic" OR Chad OR Comoros OR "Democratic Republic of the Congo" OR "Republic of the Congo" OR "Cote d'Ivoire" OR Djibouti OR Egypt OR "Equatorial Guinea" OR Eritrea OR Eswatini OR Ethiopia OR Gabon OR Gambia OR Ghana OR Guinea OR "Guinea-Bissau" OR Kenya OR Lesotho OR Liberia OR Libya OR Madagascar OR Malawi OR Mali OR Mauritania OR Mauritius OR Morocco OR Mozambique OR Namibia OR Niger OR Nigeria OR Rwanda OR "Sao Tome and Principe" OR Senegal OR Seychelles OR "Sierra Leone" OR Somalia OR "South Africa" OR "South Sudan" OR Sudan OR Tanzania OR Togo OR Tunisia OR Uganda OR Zambia OR Zimbabwe)
#4	#1 and #2 and #3
#5	Limit #4 to yr="2001 - 2023"

Web of science

#1 Dyslipidemia	Topic=(Dyslipidemia* OR dyslipid* OR hyperlipidemia* OR hyperlipid* OR "lipid disorder*" OR "lipid metabolism disorders" OR lipid* OR "lipid profile" OR hypercholesterolemia OR hypercholesterol* OR hypertriglyceridemia OR hypertriglycerid*)
#2 Type 2 diabetes	Topic=(Diabetes OR "type 2 diabetes" OR "non-insulin-dependent diabetes mellitus" OR NIDD OR T2D OR T2DM)

#3 Africa	Topic=(Africa OR Algeria OR Angola OR Benin OR Botswana OR "Burkina Faso" OR Burundi OR "Cabo Verde" OR Cameroon OR "Central African Republic" OR Chad OR Comoros OR "Democratic Republic of the Congo" OR "Republic of the Congo" OR "Cote d'Ivoire" OR Djibouti OR Egypt OR "Equatorial Guinea" OR Eritrea OR Eswatini OR Ethiopia OR Gabon OR Gambia OR Ghana OR Guinea OR "Guinea-Bissau" OR Kenya OR Lesotho OR Liberia OR Libya OR Madagascar OR Malawi OR Mali OR Mauritania OR Mauritius OR Morocco OR Mozambique OR Namibia OR Niger OR Nigeria OR Rwanda OR "Sao Tome and Principe" OR Senegal OR Seychelles OR "Sierra Leone" OR Somalia OR "South Africa" OR "South Sudan" OR Sudan OR Tanzania OR Togo OR Tunisia OR Uganda OR Zambia OR Zimbabwe)
#4	#1 and #2 and #3
#5	Limit #4 to yr="2001 - 2023"

Table S2. Characteristics of included studies

First Author	Year	Country	Region	Study site	Sample	Female	Mean age	Mean DM duration	Lipid parameter ATP III			
									TC	LDL-C	HDL-C	TG
Kimando	2017	Kenya	East	Health facility	385	252	63.3 (12.1)	9.4				✓
Fasanmade	2013	Nigeria	West	Health facility	183	101	NR	NR	✓	✓	✓	✓
Obirikorang	2018	Ghana	West	Health facility	384	237	56.4 (13.1)	9.3 (7.5)			✓	✓
Lumu	2023	Uganda	East	Health facility	500	389	55.08 (8.98)	6.95 (6.38)		✓		✓
Mashele	2018	South Africa	Southern	Health facility	176	123	58.95 (11.5)	NR			✓	✓
Ogbera	2010	Nigeria	West	Health facility	603	335	60 (10.3)	7 (6.9)			✓	✓
Okafor	2012	Nigeria	West	Health facility	233	135	55.7 (11.7)	6.7 (6.3)	✓	✓		✓
Biadgo	2018	Ethiopia	East	Health facility	159	95	49.8 (8.7)	NR	✓		✓	✓
Awadalla	2017	Sudan	North	Health facility	424	209	NR	NR		✓		✓
Adinortey	2011	Ghana	West	Health facility	288	188	55.1 (0.86)	4	✓	✓	✓	✓
Pitso	2021	South Africa	Southern	Health facility	143	92	NR	NR			✓	✓
Omodanisi	2020	South Africa	Southern	Health facility	100	50	NR	NR				✓
Haile	2020	Ethiopia	East	Health facility	248	129	49.6 (13.3)	NR	✓	✓		✓
Jisieike-Onuigbo	2011	Nigeria	West	Health facility	108	51	57.07 (11.45)	7.97 (6.73)	✓			✓
Daya	2016	South Africa	Southern	Health facility	200	114	55.89 (12.52)	NR				✓
Anto	2019	Ghana	West	Health facility	215	129	NR	NR	✓	✓	✓	✓
Achila	2020	Eritrea	East	Health facility	309	146	57.8 (11.5)	12.1 (7.4)	✓	✓	✓	✓
Gebreyesus	2022	Ethiopia	East	Health facility	421	226	58.2 (11)	NR	✓	✓	✓	✓
Teka	2019	Ethiopia	East	Health facility	115	NA	59.6 (10.2)	9 (6.9)	✓	✓		✓
Adu	2019	Ghana	West	Health facility	324	246	57.0 (7.22)	4	✓		✓	✓
Rahmoun	2019	Algeria	North	Health facility	100	63	58.47	NR	✓			✓
ELNaga	2020	Egypt	North	Health facility	100	82	NR	NR			✓	✓

Agboghoroma	2020	Nigeria	West	Health facility	200	83	56.9 (11.8)	8.7 (6.8)	✓	✓	✓	✓
Letta	2022	Ethiopia	East	Health facility	879	493	52.7 (13.3)	NR	✓			✓
Bello-Ovosi	2019	Nigeria	West	Health facility	322	161	53.5 (10.8)	NR	✓	✓	✓	✓
Kebede	2021	Ethiopia	East	Health facility	327	150	53 (17)	NR	✓	✓		✓
Almobarak	2015	Sudan	North	Health facility	167	89	NR	NR		✓	✓	✓
Nsiah	2015	Ghana	West	Health facility	150	100	51.31 (0.97)	NR			✓	✓
Ikem	2022	Nigeria	West	Health facility	400	210	60.6 (9.93)	NR		✓	✓	✓
Otieno	2020	Kenya	East	Health facility	385	252	63.3	NR				✓
Sarfo-Kantanka	2018	Ghana	West	Health facility	780	450	57.4 (9.4)	9.8 (5.6)			✓	✓
Lokpo	2022	Ghana	West	Health facility	210	114	49.98 (7.90)	NR	✓	✓	✓	✓
Alıcı	2022	Somalia	East	Health facility	529	352	51.9 (12.2)	NR	✓	✓	✓	✓
Osei-Yeboah	2017	Ghana	West	Health facility	162	101	56.42 (10.64)	NR			✓	✓
Udenze	2013	Nigeria	West	Health facility	100	56	57.5 (10.8)	NR				✓
Thuita	2019	Kenya	East	Health facility	153	91	56.07	NR	✓	✓	✓	✓
Sobngwi	2012	Multi	Multi	Health facility	2352	1437	53.0 (16.0)	8.0 (6.0)	✓			✓
Chanda	2010	Zambia	Southern	Health facility	400	222	59.30 (11.13)	NR				✓
Birarra	2018	Ethiopia	East	Health facility	256	143	NR	NR			✓	✓
Gebremeskel	2019	Ethiopia	East	Health facility	419	211	56.39 (10.18)	NR			✓	✓
Puepet	2009	Nigeria	West	Health facility	634	355	54.2 (9.1)	NR			✓	✓
Onyekwere	2011	Nigeria	West	Health facility	106	58	57.2 (9)	NR			✓	✓
Titty	2009	Ghana	West	Health facility	300	208	57.8 (11.3)	6.0 (5.0)			✓	✓
Unadike	2009	Nigeria	West	Health facility	240	134	50.8 (11)	NR				✓
Woyesa	2017	Ethiopia	East	Health facility	314	103	49.8 (9.8)	NR			✓	✓
Zerga	2020	Ethiopia	East	Health facility	330	160	NR	NR			✓	✓
Abagre	2022	Ghana	West	Health facility	430	249	58.84 (11.49)	5.30 (3.84)			✓	✓
Shita	2023	Ethiopia	East	Health facility	204	92	51.75 (11.66)	NR			✓	✓

Chamba	2017	Tanzania	East	Health facility	119	59	58.1(12.2)	9.4 (6.6)	✓	✓		✓
Bawah	2020	Ghana	West	Health facility	125	89	59.9 (9.8)	NR	✓	✓	✓	✓
Alami	2022	Morocco	North	Health facility	505	430	57.27 (10.74)	NR	✓			✓
Okafor	2008	Nigeria	West	Health facility	192	109	55.4 (11.3)	7.6 (6.9)	✓		✓	
Antwi-Baffour	2018	Ghana	West	Health facility	304	133	NR	NR	✓			
Alebiosu	2004	Nigeria	West	Health facility	218	90	52 (5.8)	8.5 (7.1)	✓			
Omar	2022	Sudan	North	Health facility	334	NA	NR	NR	✓			
Seyum	2010	Eritrea	East	Health facility	429	NR	57.4 (11.8)	NR	✓			
Akande	2007	Nigeria	West	Health facility	121	77	57.3 (10)	NR	✓			
Bizuayehu	2019	Ethiopia	East	Health facility	314	103	49.8 (9.8)	NR	✓	✓		
Asamoah-Boakye	2017	Ghana	West	Health facility	152	115	NR	NR	✓		✓	
Kalk	2008	South Africa	Southern	Health facility	754	410	NR	NR			✓	

NA: Not available, NR: Not reported, TC: Total cholesterol, TG: Triglycerides, LDL-C: Low-density lipoprotein cholesterol, HDL-C: High-density lipoprotein cholesterol

Table S3. Quality assessment of included studies using (JBI Checklist for Prevalence Studies)

First author (year)	Was the sample frame appropriate to address the target population?	Were study participants sampled in an appropriate way?	Was the sample size adequate?	Were the study subjects and the setting described in detail?	Was the data analysis conducted with sufficient coverage of the identified sample?	Were valid methods used for the identification of the condition?	Was the condition measured in a standard, reliable way for all participants?	Was there appropriate statistical analysis?	Was the response rate adequate, and if not, was the low response rate managed appropriately?	Score
Kimando (2017)	Yes	Yes	Unclear	Yes	Yes	Yes	Yes	Yes	Unclear	7
Fasanmade (2013)	Yes	No	Unclear	Yes	Yes	Yes	Yes	Yes	Unclear	6
Obirikorang (2018)	Yes	Yes	Yes	Yes	Yes	Yes	Yes	Yes	Yes	9
Lumu (2023)	Yes	No	Unclear	Yes	Yes	Yes	Unclear	Yes	Unclear	5
Mashele (2018)	Yes	Yes	Unclear	Yes	Yes	Yes	Yes	Yes	Yes	8
Ogbera (2010)	Yes	Unclear	Unclear	Yes	Yes	Yes	Yes	Yes	Unclear	6
Okafor (2012)	Unclear	No	Unclear	No	Yes	Yes	Unclear	Unclear	Unclear	2
Biadgo (2018)	Yes	Yes	Yes	Yes	Yes	Yes	Yes	Yes	Yes	9
Awadalla (2017)	Yes	Unclear	Unclear	Yes	Yes	Yes	Unclear	Unclear	Unclear	4
Adinortey (2011)	Yes	Yes	Yes	Yes	Yes	Yes	Yes	Yes	Yes	9
Pitso (2021)	Yes	No	Unclear	Yes	Yes	Yes	Unclear	Yes	Unclear	5
Omodanisi (2020)	Yes	Unclear	Unclear	Yes	Yes	Yes	Unclear	Unclear	Unclear	4

Haile (2020)	Yes	Unclear	Yes	Yes	Yes	Yes	Yes	Yes	Yes	8
Jisieike-Onuigbo (2011)	Yes	No	Unclear	Yes	Yes	Yes	Yes	Unclear	Unclear	5
Daya (2016)	Yes	No	Unclear	Yes	Yes	Yes	Unclear	Yes	Unclear	5
Anto (2019)	Yes	Yes	Unclear	Yes	Yes	Yes	Yes	Unclear	Unclear	6
Achila (2020)	Yes	Yes	Yes	Yes	Yes	Yes	Yes	Yes	Yes	9
Gebreyesus (2022)	Yes	Yes	Yes	Yes	Yes	Yes	Yes	Yes	Yes	9
Teka (2019)	Yes	No	Unclear	Yes	Yes	Yes	Yes	Yes	Unclear	6
Adu (2019)	Yes	Yes	Yes	Yes	Yes	Yes	Yes	Yes	Yes	9
Rahmoun (2019)	No	Unclear	Unclear	Yes	Yes	Yes	Unclear	No	Unclear	3
Abo (2020)	Yes	Unclear	Unclear	Yes	Yes	Yes	Yes	Unclear	Unclear	5
Agboghoroma (2020)	Yes	Unclear	Yes	Yes	Yes	Yes	Unclear	Yes	Yes	7
Letta (2022)	Yes	Yes	Yes	Yes	Yes	Yes	Unclear	Yes	Yes	8
Bello-Ovosi (2019)	Yes	Unclear	Unclear	Yes	Yes	Yes	Unclear	Yes	Unclear	5
Kebede (2021)	Yes	No	Yes	Yes	Yes	Yes	Yes	Yes	Yes	8
Almobarak (2015)	Yes	Unclear	Unclear	Yes	Yes	Yes	Unclear	Unclear	Unclear	4
Nsiah (2015)	Yes	Unclear	Unclear	Yes	Yes	Yes	Yes	Yes	Unclear	6
Ikem (2022)	Yes	Unclear	Yes	Yes	Yes	Yes	Unclear	Yes	Yes	7
Otieno (2020)	Yes	Yes	Yes	Yes	Yes	Yes	Yes	Yes	Yes	9

Sarfo-Kantanka (2018)	Yes	No	Unclear	Yes	Yes	Yes	Yes	Yes	Unclear	6
Lokpo (2022)	Yes	No	Yes	Yes	Yes	Yes	Yes	Yes	Yes	8
Alıcı (2022)	Yes	No	Yes	Yes	Yes	Yes	Yes	Yes	Unclear	7
Osei-Yeboah (2017)	Yes	No	Unclear	Yes	Yes	Yes	Yes	Yes	Unclear	6
Udenze (2013)	Yes	Yes	Unclear	Yes	Yes	Yes	Unclear	Yes	Unclear	6
Thuita (2019)	Yes	Unclear	Yes	Yes	Yes	Yes	Unclear	Yes	Yes	7
Sobngwi (2012)	Yes	Yes	Unclear	Yes	Yes	Yes	Unclear	Unclear	Unclear	5
Chanda (2010)	Yes	Unclear	Unclear	Yes	Yes	Yes	Unclear	Yes	Unclear	5
Birarra (2018)	Yes	Yes	Yes	Yes	Yes	Yes	Unclear	Yes	Yes	8
Gebremeskel (2019)	Yes	Yes	Yes	Yes	Yes	Yes	Unclear	Yes	Yes	8
Puepet (2009)	Yes	No	Unclear	Yes	Yes	Yes	Unclear	Yes	Unclear	5
Onyekwere (2011)	Yes	Unclear	Unclear	Yes	Yes	Yes	Yes	Yes	Unclear	6
Titty (2009)	Yes	Unclear	Unclear	Yes	Yes	Yes	Yes	Unclear	Unclear	5
Unadike (2009)	Yes	Unclear	Unclear	Yes	Yes	Yes	Unclear	No	Unclear	4
Woyesa (2017)	Yes	Yes	Yes	Yes	Yes	Yes	Yes	Yes	Yes	9
Zerga (2020)	Yes	Yes	Yes	Yes	Yes	Yes	Yes	Yes	Yes	9
Abagre (2022)	Yes	Yes	Yes	Yes	Yes	Yes	Yes	Yes	Yes	9

Shita (2023)	Yes	Unclear	Yes	Yes	Yes	Yes	Yes	Yes	Yes	8
Chamba (2017)	Yes	No	Unclear	Yes	Yes	Yes	Yes	Yes	Unclear	6
Bawah (2020)	Yes	No	Unclear	Yes	Yes	Yes	Yes	Yes	Yes	7
Alami (2022)	Yes	Unclear	Unclear	Yes	Yes	Yes	Unclear	Yes	Unclear	5
Okafor (2008)	Yes	No	Unclear	Yes	Yes	Yes	Unclear	Unclear	Unclear	4
Antwi-Baffour (2018)	Yes	Unclear	Unclear	Yes	Yes	Yes	Yes	Yes	Unclear	6
Alebiosu (2004)	Yes	Unclear	Unclear	Yes	Yes	Yes	Unclear	No	Unclear	4
Omar (2022)	Yes	Yes	Yes	Yes	Yes	Yes	Unclear	Yes	Yes	8
Seyum (2010)	Yes	Yes	Unclear	Yes	Yes	Yes	Unclear	Unclear	Unclear	5
Akande (2007)	Yes	Yes	Unclear	Yes	Yes	Yes	Yes	Unclear	Unclear	6
Bizuayehu (2019)	Yes	Yes	Yes	Yes	Yes	Yes	Yes	Yes	Yes	9
Asamoah-Boakye (2017)	Yes	Yes	Unclear	Yes	Yes	Yes	Yes	Yes	Yes	8
Kalk (2008)	Yes	No	Unclear	Yes	Yes	Yes	Yes	Yes	Unclear	6

Study	Cases	Total	Prevalence (%)	95% CI	Weight
Fasanmade (2013)	62	183	33.9	[27.1; 41.2]	3.7%
Biadgo (2018)	82	159	51.6	[43.5; 59.6]	3.6%
Adinortey (2011)	165	288	57.3	[51.4; 63.1]	3.8%
Haile (2020)	34	248	13.7	[9.7; 18.6]	3.5%
Jisieike-Onuigbo (2011)	58	108	53.7	[43.8; 63.3]	3.5%
Anto (2019)	135	215	62.8	[56.0; 69.3]	3.7%
Achila (2020)	192	309	62.1	[56.5; 67.6]	3.8%
Antwi-Baffour (2018)	133	304	43.8	[38.1; 49.5]	3.8%
Gebreyesus (2022)	148	421	35.2	[30.6; 39.9]	3.8%
Teka (2019)	17	115	14.8	[8.9; 22.6]	3.2%
Adu (2019)	168	324	51.9	[46.3; 57.4]	3.8%
Agboghoroma (2020)	33	200	16.5	[11.6; 22.4]	3.5%
Letta (2022)	362	879	41.2	[37.9; 44.5]	3.9%
Omar (2022)	55	334	16.5	[12.7; 20.9]	3.7%
Bello-Ovosi (2019)	164	322	50.9	[45.3; 56.5]	3.8%
Kebede (2021)	118	327	36.1	[30.9; 41.6]	3.8%
Seyum (2010)	186	429	43.4	[38.6; 48.2]	3.9%
Akande (2007)	52	121	43.0	[34.0; 52.3]	3.5%
Lokpo (2022)	66	210	31.4	[25.2; 38.2]	3.7%
Alıcı (2022)	319	529	60.3	[56.0; 64.5]	3.9%
Thuita (2019)	64	153	41.8	[33.9; 50.1]	3.6%
Sobngwi (2012)	851	2352	36.2	[34.2; 38.2]	4.0%
Bizuayehu (2019)	103	314	32.8	[27.6; 38.3]	3.8%
Asamoah-Boakye (2017)	63	152	41.4	[33.5; 49.7]	3.6%
Chamba (2017)	59	119	49.6	[40.3; 58.9]	3.5%
Bawah (2020)	52	125	41.6	[32.9; 50.8]	3.6%
Alami (2022)	132	505	26.1	[22.4; 30.2]	3.8%
**Common effect model**		**9745**	**40.5**	**[39.5; 41.6]**	--
**Random effects model**			**39.4**	**[34.7; 44.3]**	**100.0%**

Heterogeneity: $I^2 = 95\%$, $\tau^2 = 0.2655$, $p < 0.01$

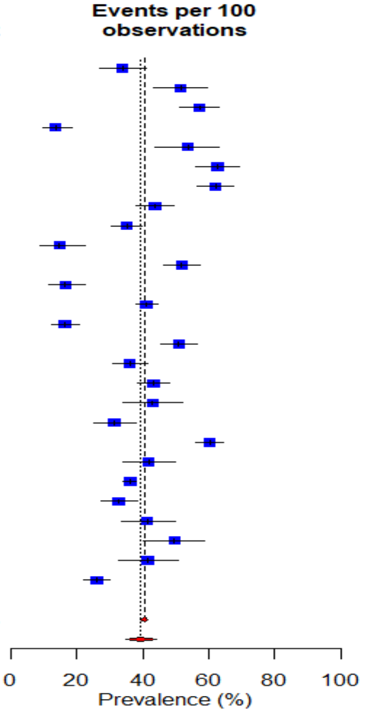

Figure S1 Forest plot for sensitivity analysis of high TC

Study	Cases	Total	Prevalence (%)	95% CI	Weight
Fasanmade (2013)	152	183	83.1	[76.8; 88.2]	5.4%
Lumu (2023)	393	500	78.6	[74.7; 82.1]	5.6%
Adinortey (2011)	182	288	63.2	[57.3; 68.8]	5.6%
Haile (2020)	71	248	28.6	[23.1; 34.7]	5.6%
Anto (2019)	102	215	47.4	[40.6; 54.3]	5.6%
Achila (2020)	236	309	76.4	[71.2; 81.0]	5.6%
Gebreyesus (2022)	181	421	43.0	[38.2; 47.9]	5.7%
Teka (2019)	45	115	39.1	[30.2; 48.7]	5.4%
Agboghoroma (2020)	99	200	49.5	[42.4; 56.6]	5.6%
Bello-Ovosi (2019)	174	322	54.0	[48.4; 59.6]	5.6%
Kebede (2021)	193	327	59.0	[53.5; 64.4]	5.6%
Ikem (2022)	146	400	36.5	[31.8; 41.4]	5.6%
Lokpo (2022)	21	210	10.0	[6.3; 14.9]	5.3%
Alıcı (2022)	427	529	80.7	[77.1; 84.0]	5.6%
Thuita (2019)	68	153	44.4	[36.4; 52.7]	5.5%
Bizuayehu (2019)	128	314	40.8	[35.3; 46.4]	5.6%
Chamba (2017)	86	119	72.3	[63.3; 80.1]	5.4%
Bawah (2020)	41	125	32.8	[24.7; 41.8]	5.4%
**Common effect model**		**4978**	**54.6**	**[53.1; 56.1]**	--
**Random effects model**			**52.5**	**[42.8; 61.9]**	**100.0%**

Heterogeneity: $I^2 = 97\%$, $\tau^2 = 0.6804$, $p < 0.01$

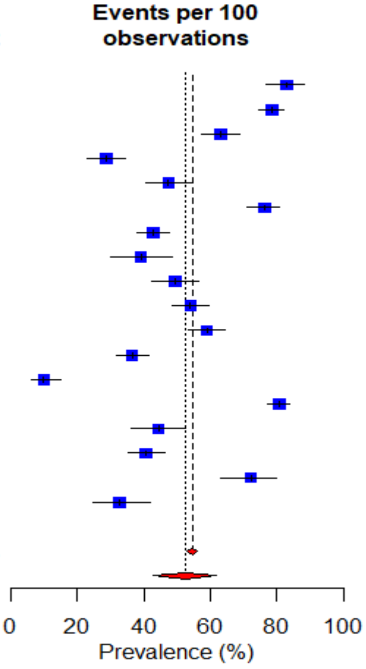

Figure S2 Forest plot for sensitivity analysis of high LDL-C

Study	Cases	Total	Prevalence (%)	95% CI	Weight
Fasanmade (2013)	113	183	61.7	[54.3; 68.8]	3.0%
Obirikorang (2018)	205	384	53.4	[48.3; 58.5]	3.1%
Mashele (2018)	105	176	59.7	[52.0; 67.0]	3.0%
Ogbera (2010)	353	603	58.5	[54.5; 62.5]	3.1%
Biadgo (2018)	50	159	31.4	[24.3; 39.3]	3.0%
Adinortey (2011)	187	288	64.9	[59.1; 70.4]	3.1%
Pitso (2021)	67	143	46.9	[38.5; 55.4]	3.0%
Anto (2019)	143	215	66.5	[59.8; 72.8]	3.0%
Achila (2020)	126	309	40.8	[35.2; 46.5]	3.1%
Gebreyesus (2022)	314	421	74.6	[70.1; 78.7]	3.1%
Adu (2019)	57	324	17.6	[13.6; 22.2]	3.0%
ELNaga (2020)	39	100	39.0	[29.4; 49.3]	2.9%
Agboghoroma (2020)	153	200	76.5	[70.0; 82.2]	3.0%
Bello-Ovosi (2019)	115	322	35.7	[30.5; 41.2]	3.1%
Nsiah (2015)	62	150	41.3	[33.4; 49.7]	3.0%
Ikem (2022)	126	400	31.5	[27.0; 36.3]	3.1%
Sarfo-Kantanka (2018)	54	780	6.9	[5.2; 8.9]	3.0%
Kalk (2008)	366	754	48.5	[44.9; 52.2]	3.1%
Lokpo (2022)	13	210	6.2	[3.3; 10.4]	2.8%
Alıcı (2022)	284	529	53.7	[49.3; 58.0]	3.1%
Osei-Yeboah (2017)	77	162	47.5	[39.6; 55.5]	3.0%
Thuita (2019)	44	153	28.8	[21.7; 36.6]	3.0%
Birarra (2018)	172	256	67.2	[61.1; 72.9]	3.1%
Gebremeskel (2019)	144	419	34.4	[29.8; 39.1]	3.1%
Puepet (2009)	444	634	70.0	[66.3; 73.6]	3.1%
Onyekwere (2011)	37	106	34.9	[25.9; 44.8]	2.9%
Titty (2009)	106	300	35.3	[29.9; 41.0]	3.1%
Woyesa (2017)	123	314	39.2	[33.7; 44.8]	3.1%
Zerga (2020)	160	330	48.5	[43.0; 54.0]	3.1%
Abagre (2022)	302	430	70.2	[65.7; 74.5]	3.1%
Shita (2023)	52	204	25.5	[19.7; 32.0]	3.0%
Asamoah-Boakye (2017)	32	152	21.1	[14.9; 28.4]	3.0%
Bawah (2020)	20	125	16.0	[10.1; 23.6]	2.9%
**Common effect model**		**10235**	**47.9**	**[46.8; 49.0]**	--
**Random effects model**			**42.6**	**[36.0; 49.4]**	**100.0%**

Heterogeneity: $I^2 = 98\%$, $\tau^2 = 0.6296$, $p < 0.01$

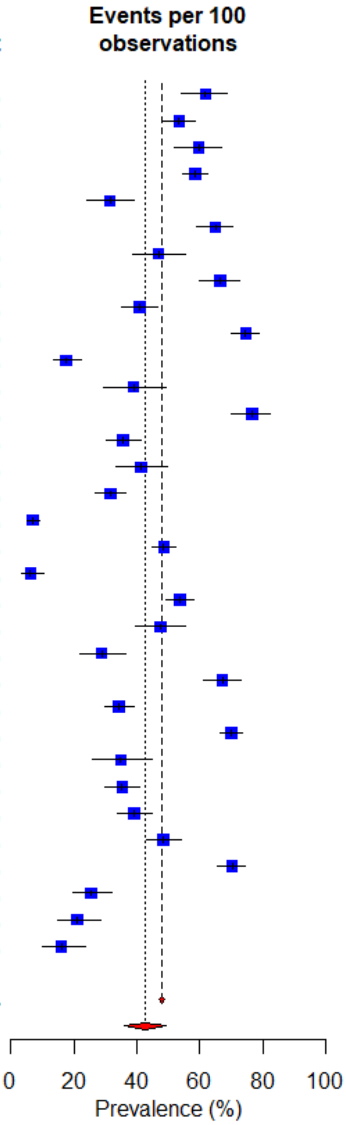

Figure S3 Forest plot for sensitivity analysis of low HDL-C

Study	Cases	Total	Prevalence (%)	95% CI	Weight
Kimando (2017)	210	385	54.5	[49.4; 59.6]	2.3%
Fasanmade (2013)	45	183	24.6	[18.5; 31.5]	2.2%
Obirikorang (2018)	178	384	46.4	[41.3; 51.5]	2.3%
Lumu (2023)	279	500	55.8	[51.3; 60.2]	2.3%
Mashele (2018)	48	176	27.3	[20.8; 34.5]	2.2%
Ogbera (2010)	36	603	6.0	[4.2; 8.2]	2.2%
Biadgo (2018)	99	159	62.3	[54.2; 69.8]	2.2%
Adinortey (2011)	119	288	41.3	[35.6; 47.2]	2.3%
Pitso (2021)	67	143	46.9	[38.5; 55.4]	2.2%
Haile (2020)	119	248	48.0	[41.6; 54.4]	2.3%
Jisieike-Onuigbo (2011)	61	108	56.5	[46.6; 66.0]	2.2%
Daya (2016)	77	200	38.5	[31.7; 45.6]	2.2%
Anto (2019)	104	215	48.4	[41.5; 55.3]	2.3%
Achila (2020)	240	309	77.7	[72.6; 82.2]	2.3%
Gebreyesus (2022)	173	421	41.1	[36.4; 46.0]	2.3%
Teka (2019)	51	115	44.3	[35.1; 53.9]	2.2%
Adu (2019)	243	324	75.0	[69.9; 79.6]	2.3%
ELNaga (2020)	47	100	47.0	[36.9; 57.2]	2.2%
Agboghoroma (2020)	53	200	26.5	[20.5; 33.2]	2.2%
Letta (2022)	555	879	63.1	[59.9; 66.3]	2.3%
Bello-Ovosi (2019)	132	322	41.0	[35.6; 46.6]	2.3%
Kebede (2021)	138	327	42.2	[36.8; 47.8]	2.3%
Nsiah (2015)	49	150	32.7	[25.2; 40.8]	2.2%
Ikem (2022)	96	400	24.0	[19.9; 28.5]	2.3%
Otieno (2020)	210	385	54.5	[49.4; 59.6]	2.3%
Sarfo-Kantanka (2018)	170	780	21.8	[18.9; 24.9]	2.3%
Lokpo (2022)	22	210	10.5	[6.7; 15.4]	2.2%
Alıcı (2022)	251	529	47.4	[43.1; 51.8]	2.3%
Osei-Yeboah (2017)	27	162	16.7	[11.3; 23.3]	2.2%
Udenze (2013)	10	100	10.0	[4.9; 17.6]	2.0%
Thuita (2019)	99	153	64.7	[56.6; 72.3]	2.2%
Sobngwi (2012)	26	2352	1.1	[0.7; 1.6]	2.2%
Chanda (2010)	125	400	31.2	[26.7; 36.0]	2.3%
Birarra (2018)	176	256	68.8	[62.7; 74.4]	2.3%
Gebremeskel (2019)	189	419	45.1	[40.3; 50.0]	2.3%
Puepet (2009)	393	634	62.0	[58.1; 65.8]	2.3%
Onyekwere (2011)	9	106	8.5	[4.0; 15.5]	2.0%
Titty (2009)	102	300	34.0	[28.7; 39.7]	2.3%
Woyesa (2017)	221	314	70.4	[65.0; 75.4]	2.3%
Zerga (2020)	170	330	51.5	[46.0; 57.0]	2.3%
Abagre (2022)	139	430	32.3	[27.9; 37.0]	2.3%
Shita (2023)	52	204	25.5	[19.7; 32.0]	2.2%
Chamba (2017)	64	119	53.8	[44.4; 63.0]	2.2%
Bawah (2020)	4	125	3.2	[0.9; 8.0]	1.7%
Alami (2022)	121	505	24.0	[20.3; 27.9]	2.3%
**Common effect model**		**15952**	**43.7**	**[42.8; 44.6]**	**--**
**Random effects model**			**37.0**	**[31.3; 43.0]**	**100.0%**

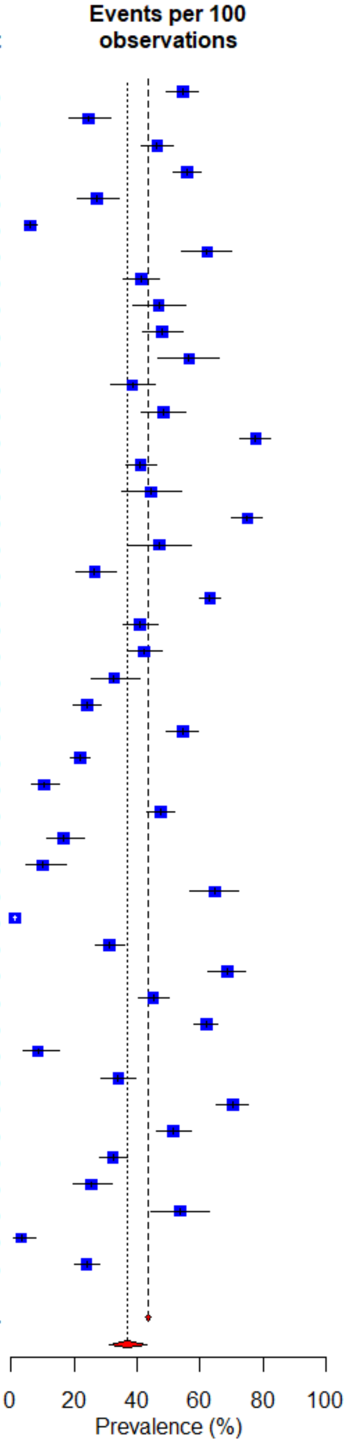

Heterogeneity: $I^2 = 98\%$, $\tau^2 = 0.7238$, $p = 0$

Figure S4 Forest plot for sensitivity analysis of high TG

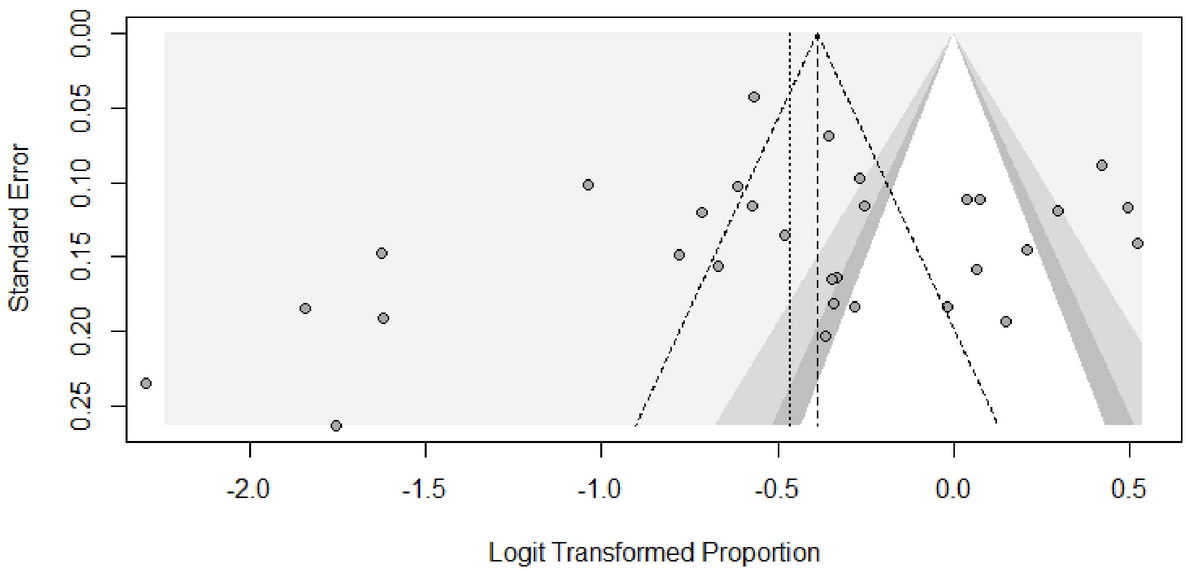

Figure S5 Funnel plot prevalence of high TC in persons with T2D

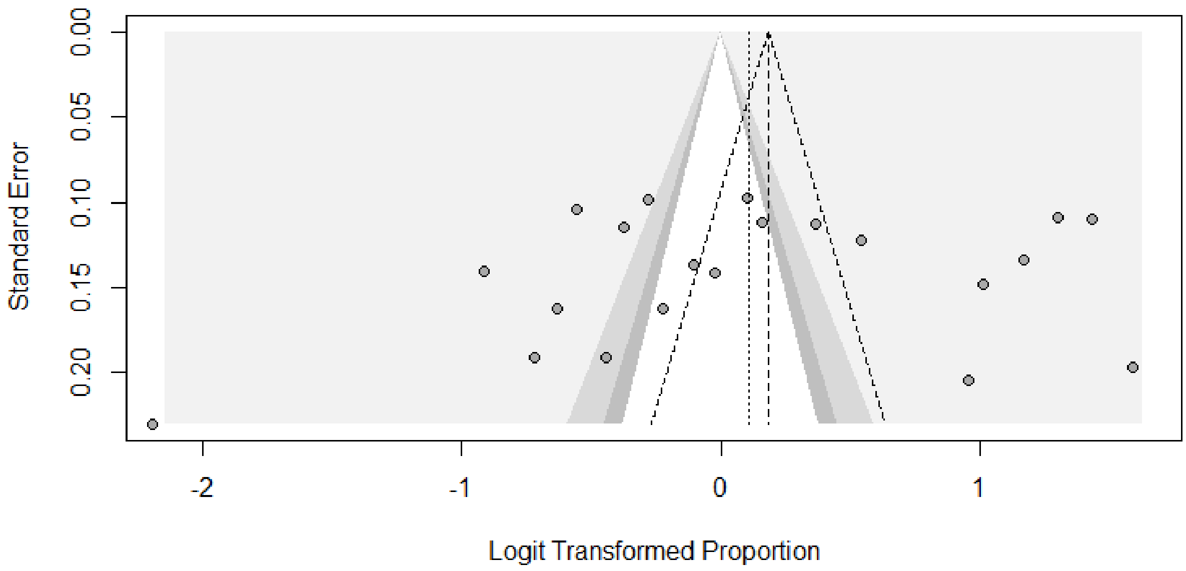

Figure S6 Funnel plot prevalence of high LDL-C in persons with T2D

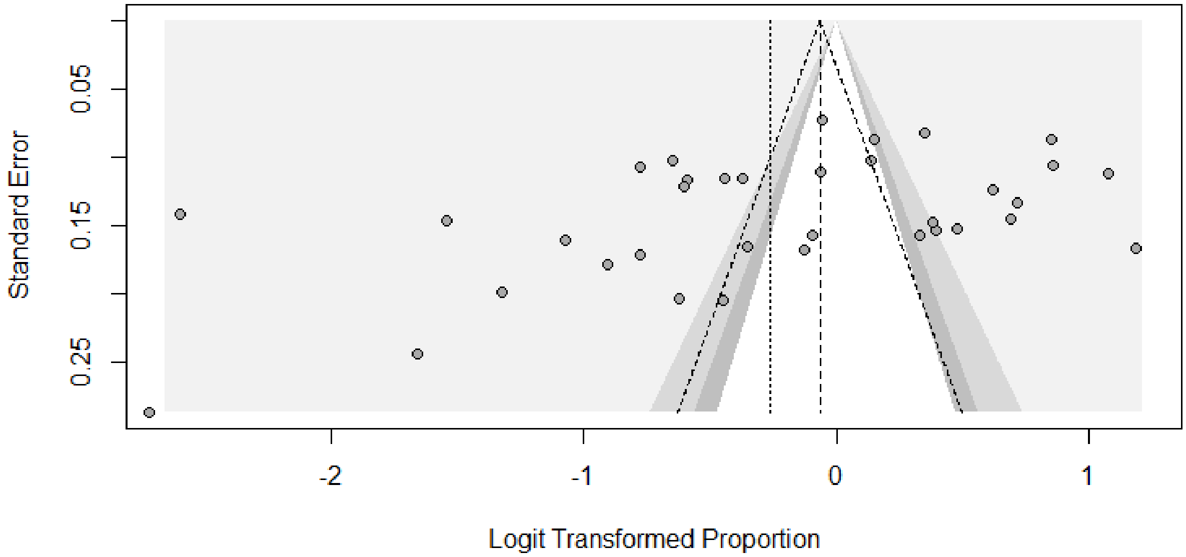

Figure S7 Funnel plot prevalence of low HDL-C in persons with T2D

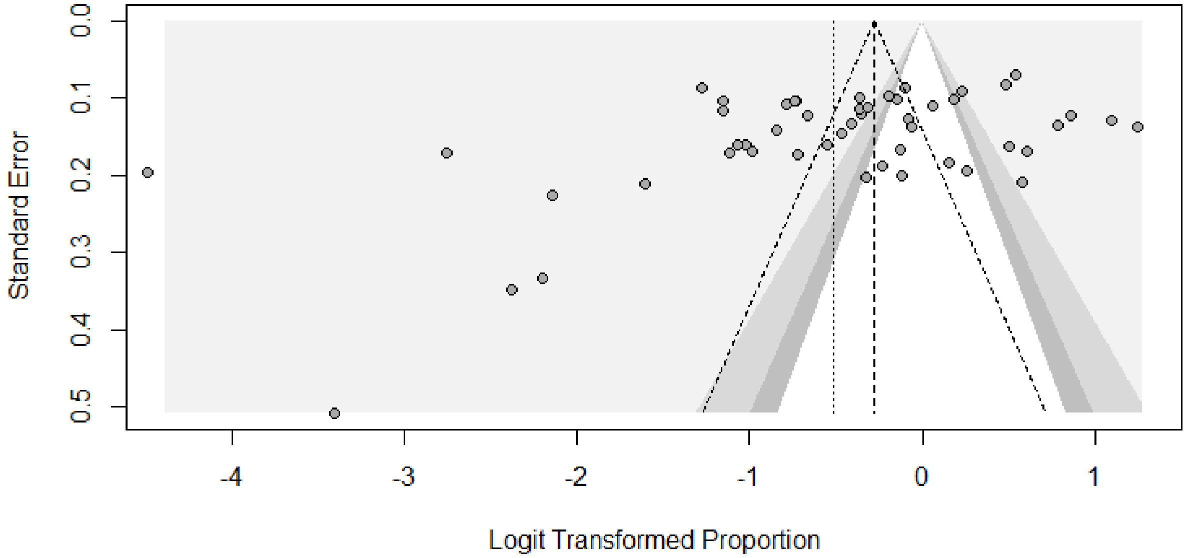

Figure S8 Funnel plot prevalence of high TG in persons with T2D
